# MST-YOLO: Small Object Detection Model for Autonomous Driving

**DOI:** 10.3390/s24227347

**Published:** 2024-11-18

**Authors:** Mingjing Li, Xinyang Liu, Shuang Chen, Le Yang, Qingyu Du, Ziqing Han, Junshuai Wang

**Affiliations:** 1 College of Electronic Information Engineering, Changchun University, Changchun 130022, China; limj@ccu.edu.cn (M.L.); 19935468525@163.com (X.L.); 13663846625@163.com (L.Y.); duyu760544@163.com (Q.D.); hzqqqz@163.com (Z.H.); 13283063641@163.com (J.W.); 2 School of Information and Electronic Engineering, Shangqiu Institute of Technology, Shangqiu 476000, China

**Keywords:** autonomous driving, YOLOv8 algorithm, small object detection

## Abstract

Autonomous vehicles operating in public transportation spaces must rapidly and accurately detect all potential hazards in their surroundings to execute appropriate actions such as yielding, lane changing, and overtaking. This capability is a prerequisite for achieving advanced autonomous driving. In autonomous driving scenarios, distant objects are often small, which increases the risk of detection failures. To address this challenge, the MST-YOLOv8 model, which incorporates the C2f-MLCA structure and the ST-P2Neck structure to enhance the model’s ability to detect small objects, is proposed. This paper introduces mixed local channel attention (MLCA) into the C2f structure, enabling the model to pay more attention to the region of small objects. A P2 detection layer is added to the neck part of the YOLOv8 model, and scale sequence feature fusion (SSFF) and triple feature encoding (TFE) modules are introduced to assist the model in better localizing small objects. Compared with the original YOLOv8 model, MST-YOLOv8 demonstrates a 3.43% improvement in precision (P), an 8.15% improvement in recall (R), an 8.42% increase in mAP_0.5, a reduction in missed detection rate by 18.47%, a 70.97% improvement in small object detection AP, and a 68.92% improvement in AR.

## 1. Introduction

Autonomous driving refers to the technology and systems that enable a vehicle to perceive its environment and make decisions independently, using artificial intelligence, computer vision, and sensor technologies to ensure safe driving. Over the past two decades, the field of object detection has seen breakthrough advancements, primarily divided into two directions: traditional object detection algorithms and deep learning-based object detection algorithms.

In early driving scene detection, methods commonly relied on manually extracted features. The histogram of oriented gradients (HOG) detector, proposed in 2005 [1], improved detection accuracy by calculating overlapping local contrast normalization on a dense grid of uniformly spaced cells. This approach demonstrated stability in handling local object deformations and varying lighting conditions, laying a strong foundation for subsequent detection methods. However, it struggled with detecting occluded objects. The deformable parts model (DPM), introduced in 2008 [2], consisted of a root filter and multiple part filters. It improved detection precision through techniques such as hard negative mining, bounding box regression, and context priming. While DPM was fast and capable of adapting to object deformations, it performed poorly with large-scale rotations, leading to stability issues.

Deep learning-based object detection algorithms can be divided into two categories based on their process characteristics: two-stage object detection algorithms and one-stage object detection algorithms. In two-stage object detection, image segmentation algorithms are first used to extract candidate regions, and then, these regions are fed into a convolutional neural network (CNN) through a sliding window for classification and regression tasks. The advantage of this approach lies in its ability to achieve precise object classification and localization by fully extracting features. However, the downside is the slow processing speed. The R-CNN family of algorithms is representative of two-stage detection algorithms [3,4,5,6].

One-stage object detection algorithms, on the other hand, treat object classification and localization as a regression problem. By inputting the entire image into the network, the position and class information of the bounding box are directly regressed at the output layer. This method transforms the object detection task into a regression problem, significantly improving detection speed. The strengths of such algorithms include a simpler network structure and faster detection speed, making them highly suitable for real-time applications. However, compared with two-stage detection algorithms, they tend to have lower detection accuracy. The single-shot multi-box detector (SSD) series and the You Only Look Once (YOLO) series are typical representatives of one-stage object detection algorithms [7,8]. In recent years, DETR series algorithms such as Deformable detr, Dn-detr, and DQ-detr, have gradually emerged [9,10,11]. However, this series of algorithms requires a lot of time for training, has slow detection speed, and poor real-time performance, making them unsuitable for use in the field of autonomous driving. Therefore, most researchers adopt the YOLO series algorithm to complete the task of detecting small targets in autonomous driving scenarios.

While two-stage detection algorithms have a more complex network structure and slower detection speeds, one-stage detection algorithms offer a simpler structure and faster detection, making them more suitable for object detection tasks. To meet the speed requirements of dynamic object detection in autonomous driving, YOLOv8 is selected as the base network for dynamic object detection in this paper. Several structural optimizations were made on the original YOLOv8 model to better suit the specificity of real-time detection. The contributions of this paper can be summarized as follows:

1. To enhance the model’s ability to detect small objects, the mixed local channel attention (MLCA) mechanism is introduced to the C2f structure in this paper, allowing the model to focus more on regions containing small objects.
2. In the neck section of the YOLOv8 model, a P2 detection layer is added, along with the integration of the scale sequence feature fusion (SSFF) and triple feature encoding (TFE) modules, which assist the model in improving the localization of small objects.

## 2. Related Works

### 2.1. YOLO Object Detection Algorithm

Convolutional neural networks (CNNs) emerged in 2012, revolutionizing the field of object detection and elevating it to new heights. Based on the computational process, CNN-based object detection algorithms can be categorized into one-stage and two-stage approaches. Although one-stage detection algorithms operate faster, two-stage methods generally offer higher accuracy. The first one-stage detection technique was YOLOv1 [12]. This method divides an image into a grid and simultaneously predicts the position of the bounding boxes and the corresponding class probabilities for each grid. Despite its speed of 155 frames per second, YOLOv1 was slower compared with other methods, such as two-stage approaches and demonstrated poor performance in detecting small objects.

YOLOv2 replaced the backbone feature extraction network with Darknet-19, which reduced the number of convolution operations compared with YOLOv1, thereby decreasing computational complexity. For classification tasks, YOLOv2 employed a joint training technique combining object detection and classification, using methods like Word Tree to improve detection accuracy, speed, and the number of recognizable categories. However, YOLOv2 still struggled with accuracy issues in detecting objects of varying sizes, particularly small objects.

The most significant change in YOLOv3 was the introduction of feature pyramid networks (FPN) and the use of three detection branches to detect objects of different sizes, thus improving detection accuracy [13]. YOLOv4 built upon the overall structure of YOLOv3 and incorporated several advanced deep learning techniques, such as data augmentation, self-adversarial training, and the addition of the spatial pyramid pooling (SPP) module, significantly improving detection accuracy while maintaining the same speed [14].

YOLOv5 introduced further optimizations to YOLOv4, adding a focus layer to accelerate training speed and incorporating the CSP (cross-stage partial) module into the neck structure, replacing the SPP with the SPPF (spatial pyramid pooling—fast) structure [15]. YOLOv8, developed as a further enhancement by the YOLOv5 team, introduced the C2f module in the backbone, which integrates advanced features and contextual information to improve detection accuracy. Additionally, it used CIoU and DFL loss functions to enhance performance, especially in detecting small objects. While YOLOv8 offers significantly higher accuracy than YOLOv5, it comes with a slight decrease in speed.

YOLOv8 includes five models: n, s, m, l, and x. YOLOv8n is the smallest and fastest model, whereas YOLOv8x is the most accurate but the slowest. To balance detection speed and accuracy, the YOLOv8s model is selected as the base network for further development in this paper.

### 2.2. Small Object Detection

The application of deep learning techniques has led to the latest advancements in general object detection. However, detecting small objects in images remains a complex challenge due to their limited size, subtle appearances, and intricate geometric cues. Enhancing small object detection capabilities is of significant importance in practical applications such as underwater target detection, autonomous driving, and drone surveillance.

Current trends for improving small object detection include multi-scale feature extraction, the introduction of attention mechanisms, lightweight network design, data augmentation, and transfer learning. Small objects often have a limited size in images, requiring effective multi-scale feature extraction. Using convolutional layers with different receptive fields or networks that incorporate pyramid structures can effectively capture target information at various scales. For instance, the feature pyramid network (FPN) algorithm utilizes both low-level features with high resolution and high-level features with rich semantic information simultaneously [16]. By fusing features from different layers, it achieves efficient predictions.

Leveraging the relationship between objects and their surroundings is another effective approach to improve small object detection accuracy. Attention mechanisms, inspired by cognitive attention in artificial neural networks, enhance the importance of certain parts of the input data while reducing the importance of others based on context. Examples of these mechanisms include self-attention and channel attention mechanisms [17,18].

Given the high computational and storage demands of small object detection tasks, researchers have proposed lightweight network structures, such as MobileNet and EfficientNet [19], which reduce computational and storage overhead while maintaining detection accuracy. To address the issue of data scarcity in small object detection, researchers also employ techniques like data augmentation and transfer learning to increase the amount of training data and enrich data distribution, thus improving the generalization capability of small object detection algorithms.

Currently, deep learning-based small object detection has found numerous applications. In our work, we enhance small object detection by integrating two key techniques: the introduction of the MLCA attention module, which leverages the advantages of both local and channel attention, helping the model learn more discriminative feature representations and improving its generalization ability. Additionally, we introduce the SSFF and TFE modules to assist the model in better localizing small objects.

## 3. Proposed Algorithm

### 3.1. Network Structure of MST-YOLOv8

**Figure 1:**
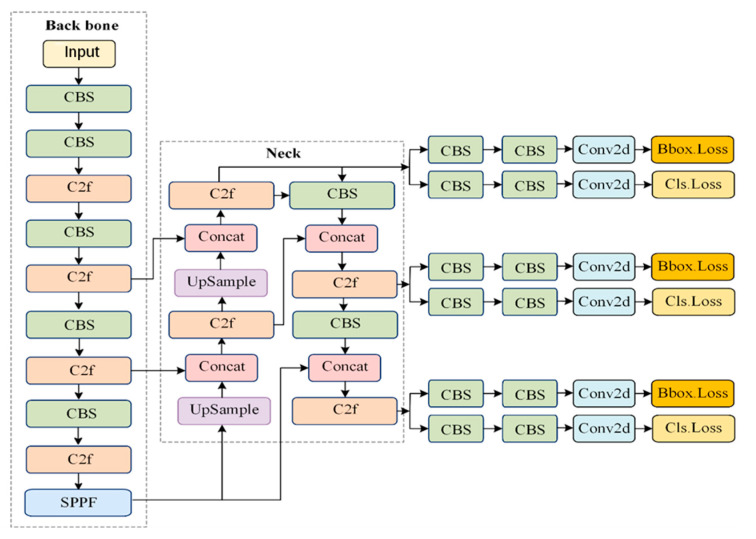
Model structure of YOLOv8.

Figure 1 illustrates the structure of the YOLOv8 model, which is largely similar to YOLOv5 in the backbone, with the main difference being the replacement of the C3 module with the C2f module. The design of the C2f module is inspired by the ELAN concept from YOLOv7 [20], which merges the C3 module with the ELAN module to form C2f. This allows YOLOv8 to capture more diverse gradient flow information. At the end of the backbone, the SPPF module is employed where three 5 × 5 Maxpool layers are sequentially passed through and concatenated to ensure accuracy in detecting objects at different scales.

In the neck section, YOLOv8 uses the PAN-FPN feature fusion method, which enhances the integration and utilization of feature information across different scales. For the head section, YOLOv8 incorporates the decoupled head idea from YOLOx [21], combining confidence scores and bounding box regression to achieve higher precision levels. The YOLOv8 algorithm is relatively well-rounded in various aspects, but there are still challenges in detecting small objects in complex scenes. The inaccuracy in detecting small objects can be attributed to two main reasons. First, during feature extraction, small objects are often overshadowed by larger objects, leading to a lack of sufficient small object information in the deeper layers. This causes the network to overlook small objects throughout the learning process, resulting in poor detection performance. Second, small objects are more difficult to distinguish and localize in images compared with normal-sized objects.

**Figure 2:**
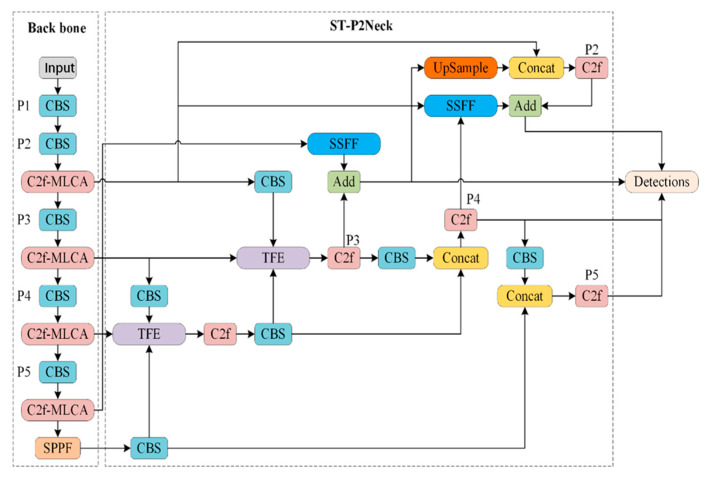
MST-YOLOv8 structure diagram.

To address these issues, the MST-YOLOv8 network model proposed in this paper significantly improves the detection of small objects while maintaining the performance of detecting normal-sized objects. The structure of the MST-YOLOv8 network model is shown in Figure 2.

### 3.2. C2f-MLCA Structure

#### 3.2.1. C2f Module of YOLOv8

**Figure 3:**
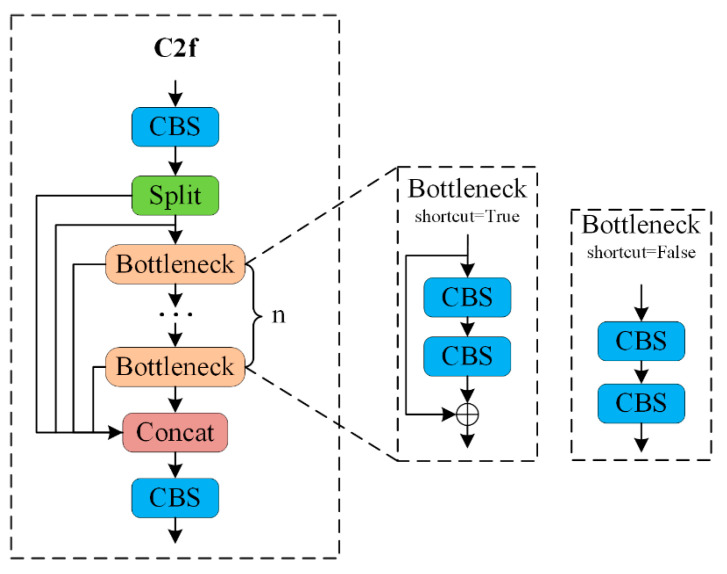
C2f Module structure diagram.

In YOLOv8, the C2f module plays a crucial role in enhancing the model’s accuracy. The structure of the C2f module, which employs the Bottleneck design concept, is depicted in Figure 3. This design divides the feature maps along Dimension 1 into two parts, improving the model’s capacity for nonlinear representation to better handle complex image features.

By incorporating the C2f module, the model becomes more adept at capturing intricate features within images, leading to improved performance in object detection tasks. Additionally, the C2f module offers substantial scalability, allowing for performance enhancement without significantly increasing computational costs.

However, in the C2f module, it is essential to fuse feature maps from different stages to provide comprehensive information. When merging features, local information from various regions must be considered. To address these challenges, the mixed local channel attention (MLCA) module is integrated into the C2f structure in this paper, enhancing its ability to process and fuse features more effectively.

#### 3.2.2. Mixed Local Channel Attention

**Figure 4:**
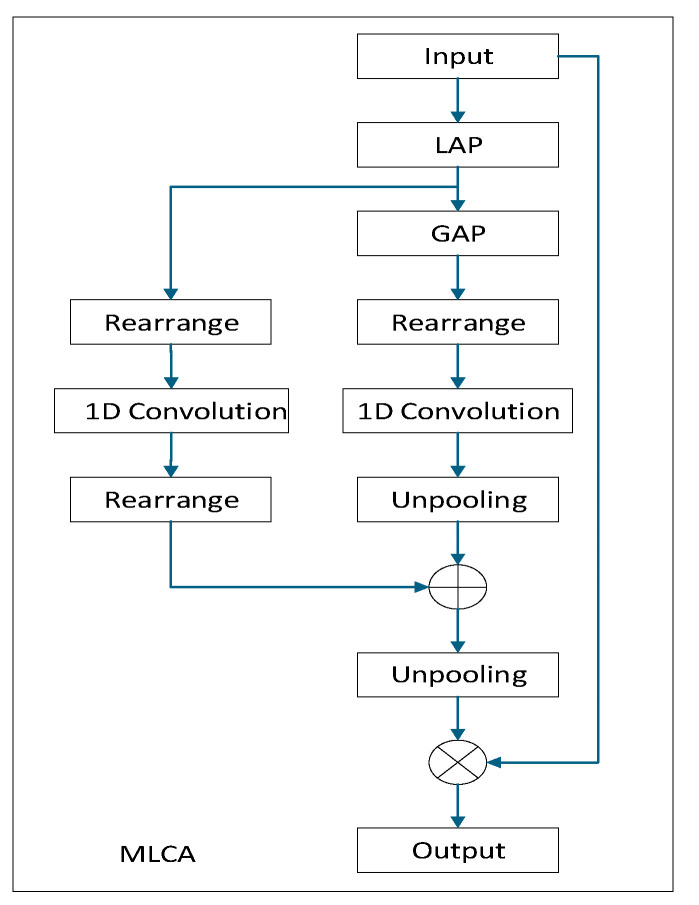
MLCA structure diagram.

The principle of MLCA is illustrated in Figure 4 [22]. The input feature vector of MLCA undergoes two pooling steps. Initially, the input is converted into a 1 × C × ks × ks vector to extract local spatial information through the first local pooling. Based on this initial stage, the input is split into two branches: the first branch captures global information, while the second branch retains local spatial information. After a 1D convolution, the two vectors are restored to their original resolution through unpooling, followed by information fusion to achieve mixed attention.

By introducing the mixed local channel attention (MLCA) mechanism, channel attention is incorporated into the fusion process. This helps assign different weights to features across various channels, enhancing the feature representation capabilities and improving the effectiveness of feature fusion. The local attention mechanism aids the model in focusing more on important local regions, thereby increasing its sensitivity to local information, which is crucial for improving object detection performance. MLCA leverages the advantages of both local and channel attention, allowing the model to learn more distinctive feature representations and improving its generalization ability.

**Figure 5:**
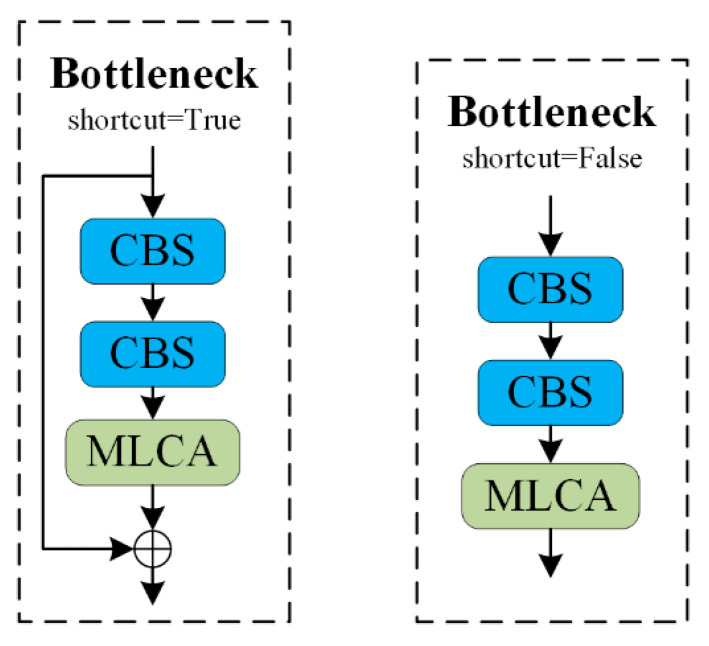
C2f-MLCA structure diagram.

The modified bottleneck structure within the improved C2f module is shown in Figure 5. Initially, the input is passed through two convolution layers, and the result is fed into the MLCA attention module. If the shortcut is set to True, the input is added to the output of the MLCA module to form the final output. If the shortcut is set to False, the output from the MLCA module is used directly as the final output.

By incorporating the MLCA attention mechanism into the bottleneck structure, the model’s feature representation capability and the importance weighting of features are significantly enhanced, allowing it to focus more effectively on regions with small objects. The inclusion of the attention mechanism within the bottleneck enables the model to flexibly learn the relationships between features, thereby improving overall model performance.

### 3.3. ST-P2Neck Structure

#### 3.3.1. The Neck Section of YOLOv8

**Figure 6:**
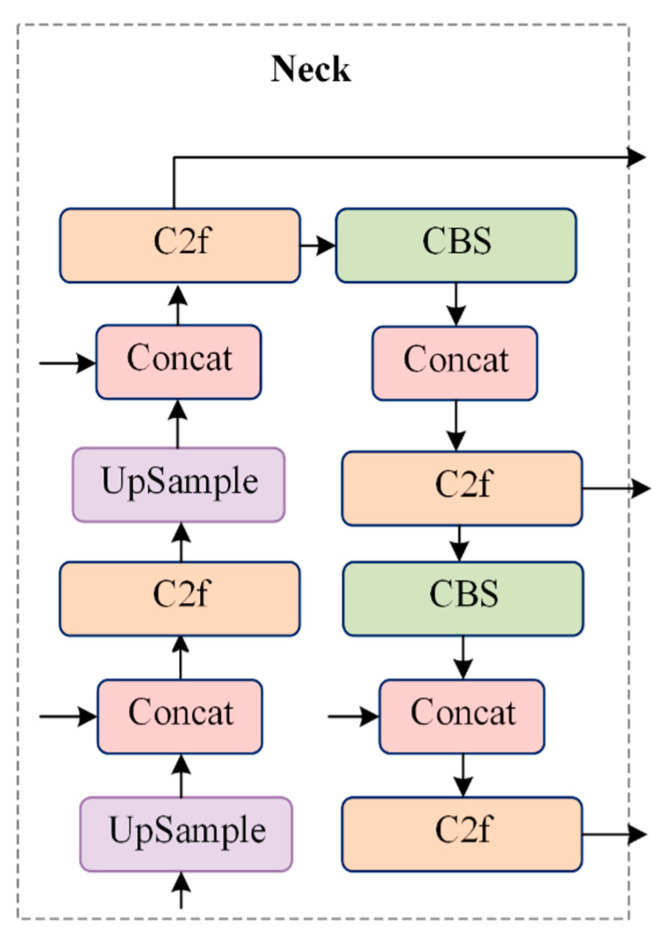
Neck structure diagram of YOLOv8.

As shown in Figure 6, the neck section of YOLOv8 employs multi-scale feature fusion to combine features from different layers of the network. The upper layers capture more detailed information due to the increased depth of the network, while the lower layers retain positional information as a result of having fewer convolutional layers.

Inspired by YOLOv5, FPN performs top-down upsampling to enrich the feature information in the lower feature maps, while PAN conducts bottom-up downsampling to capture more information from the top feature maps. The outputs of these two processes are fused to ensure precise predictions across various object sizes. In the YOLOv8 model, the feature pyramid path aggregation network (FP-PAN) [23] is employed, and convolution operations in the upsampling process are removed to reduce computational costs.

#### 3.3.2. Scale Sequence Feature Fusion

For the multi-scale problem of dynamic objects in autonomous driving scenarios, feature pyramid structures have been commonly employed for feature fusion where only summation or concatenation is used to merge pyramid features. However, these conventional feature pyramid networks fail to effectively leverage the correlations between all pyramid feature maps. To address this, Ming Kang et al. proposed a novel scale sequence feature fusion (SSFF) method that better integrates high-dimensional information from deeper feature maps with detailed information from shallower ones [24]. In this approach, the image size changes during downsampling, but scale-invariant features remain consistent. The scale space is constructed along the scale axis of the image, representing not just one scale, but a range of possible scales an object can have. Scale refers to the level of detail in an image—blurry images may lose fine details but can retain the structural features of the image. The mathematical expression for this is as follows:

(1) $$F_{\sigma }\left(w,h\right)=G_{\sigma }\left(w,h\right)\times f\left(w,h\right)$$

(2) $$G_{\sigma }\left(w,h\right)=\frac{1}{2\pi \sigma^{2}}e^{-\frac{w^{2}+h^{2}}{2\sigma^{2}}}$$

In this expression, *f*(*w*, *h*) represents a two-dimensional input image with width w and height h. *F_σ_*(*w*, *h*) is generated by a series of convolutions using a two-dimensional Gaussian filter *G_σ_*(*w*, *h*) for smoothing. *σ* denotes the scaling parameter of the standard deviation used in the two-dimensional Gaussian filter during the convolution process.

The generated images maintain the same resolution but differ in scale. Thus, feature maps of varying sizes can be treated as a scale space, and feature maps with different resolutions can be adjusted to the same resolution for concatenation. Inspired by the use of 2D and 3D convolutional operations across multiple video frames, the feature maps at different scales are horizontally stacked, and 3D convolution is applied to extract scale sequence features from these maps. Since the high-resolution feature map at level P3 contains most of the crucial information for small object detection and segmentation, the SSFF module is designed based on the P3 level.

**Figure 7:**
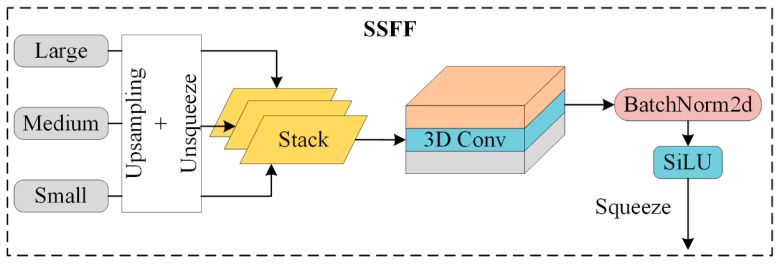
SSFF module structure diagram.

As shown in Figure 7, the SSFF module’s structure first uses 1 × 1 convolution to adjust the channel numbers of the P4 and P5 feature levels to 256. Then, the nearest neighbor interpolation method is applied to resize the P4 and P5 feature maps to match the size of the P3 level. The unsqueeze method is used to add a new dimension to each feature layer, and the adjusted P3, P4, and P5 tensors are concatenated along the third dimension, resulting in a new tensor named “combine”. This “combine” tensor is then passed through a 3D convolution layer, and the output is fed into a 3D batch normalization layer for normalization. Afterward, the normalized output is passed through the SiLU activation function for activation. Finally, the tensor is compressed along the second dimension, completing the scale sequence feature extraction process.

Integrating the SSFF module into the neck enables the fusion of feature maps from different scales, allowing the model to simultaneously focus on multi-scale information. This enhances the model’s ability to learn feature representations of targets at varying scales, thereby improving its detection capabilities across different-sized objects. Consequently, this boosts the accuracy and robustness of object detection.

#### 3.3.3. Triple Feature Encoding

To accurately identify densely overlapping small objects, the image can be enlarged to reference and compare shape or appearance variations across different scales. Since the different feature layers of the backbone network have varying sizes, traditional FPN fusion mechanisms only upsample small-sized feature maps and, then, split or add them to the feature map of the preceding layer, thereby overlooking the rich detailed information from larger feature layers. To address this issue, the triple feature encoding (TFE) module is proposed. This method divides features of large, medium, and small sizes, incorporates large-sized feature maps, and amplifies the features to enhance the detailed feature information.

**Figure 8:**
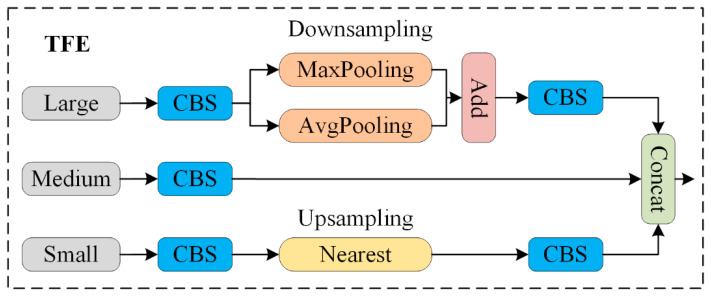
TEE module structure diagram.

In Figure 8, the structure of the triple feature encoding (TFE) module is illustrated. Before feature encoding, it is essential to adjust the number of feature channels to ensure consistency with the main scale features. After processing the large-sized feature map (large) through a convolution module, its channel count is adjusted to 1C. Then, a hybrid structure of max pooling and average pooling is applied for downsampling, which helps retain the high-resolution features and preserves the effectiveness and diversity of the image. For the small-sized feature map (small), a convolution module is also used to adjust the channel count, followed by upsampling using the nearest neighbor interpolation method. This approach helps maintain the richness of local features in the low-resolution image, preventing the loss of small object feature information. Finally, the large, medium, and small feature maps, now of the same size, undergo a final convolution and are concatenated along the channel dimension. The specific expression for this process is as follows:

(3) $$F_{TFE}=\mathrm{Concat}\left(F_{1},F_{m},F_{s}\right)$$

In this expression, $F_{TFE}$ represents the feature map output by the TFE module. $F_{1}$, $F_{m}$, and $F_{s}$ represent the large, medium, and small-sized feature maps, $F_{TFE}$ is formed by concatenating $F_{1}$, $F_{m}$, and $F_{s}$. The resolution of $F_{TFE}$ is the same as that of $F_{m}$, and its number of channels is three times that of $F_{m}$.

By integrating the TFE module into the neck, the model can encode spatial information from feature maps, enabling it to better understand the spatial position and shape of the objects. This enhancement improves the accuracy of object detection.

## 4. Experiments

### 4.1. Experimental Environment

The proposed MST-YOLOv8 algorithm was executed on a Windows 10 operating system with an Intel(R) Xeon(R) CPU E5-2680v4, equipped with a 14-core configuration. The Intel(R) Xeon(R) CPU E5-2680v4 is manufactured by Intel Corporation, which is headquartered in Santa Clara, CA, USA. The GPU used was a 3080 Ti with 12 GB of memory, and the GPU driver version was 535.129.03. The system had 32 GB of RAM. The CUDA version utilized was 11.6.0, while the PyTorch version was 1.13.1, and the Python version was 3.8.

### 4.2. Dataset

We evaluated and validated the generalization performance of our model using two challenging object detection datasets.

#### 4.2.1. SODA-10M

The SODA-10M dataset, jointly released by Huawei Noah’s Ark Lab and Sun Yat-sen University in 2021, is a next-generation 2D autonomous driving dataset characterized by its large scale, strong diversity, and robust generalization capabilities. It primarily annotates categories related to pedestrian, cyclist, car, truck, and tram scenarios for autonomous vehicles to handle various situations. The dataset includes a range of road scenes (urban, highway, rural, and park), weather conditions (clear, cloudy, rainy, and snowy), and times of day (daytime, nighttime, and dawn/dusk). The diversity in scenes, weather, and time periods ensures its effectiveness as a self-supervised pre-training dataset and as semi-supervised additional data for generalizing performance in downstream autonomous driving tasks.

We restructured the SODA-10M dataset into new subsets: 7000 images for training, 2000 images for validation, and 1000 images for testing. Additionally, we converted the annotation format from the original JSON files to the TXT file format required by YOLO.

#### 4.2.2. BDD100K

The BDD100K dataset, released by the AI Lab at the University of California, Berkeley in 2018, is one of the publicly available driving datasets. The dataset includes videos collected from various locations across the United States, covering different times, weather conditions (including sunny, cloudy, and rainy weather, as well as day and night), and driving scenarios. The geographic locations from which the data were collected include New York, Berkeley, and San Francisco. In this dataset, road object detection is annotated with 2D bounding boxes for categories such as buses, traffic lights, traffic signs, people, bicycles, trucks, motorcycles, cars, trains, and passengers, across 100,000 images [25]. We extracted 1000 images from the BDD100K dataset for practical testing to evaluate the model’s generalization capability.

### 4.3. Evaluation Metrics

Three evaluation metrics were used in this experiment to evaluate the performance of the algorithm.

Precision is the percentage of samples predicted to be positive that are actually positive. The formula is as follows:

(4) $$Precision\left(classes\right)=\frac{TP}{TP+FP}$$

where TP indicates that positive samples are predicted to be positive and FP indicates that negative samples are predicted to be positive.

Recall is the percentage of all positive samples that are actually predicted to be positive. The formula is as follows:

(5) $$Recall\left(classes\right)=\frac{TP}{TP+FN}$$

where FN indicates that a positive sample is predicted to be a negative sample.

mAP is the average category AP, which is the AP of all categories divided by the total number of categories. The formula is as follows:

(6) $$mAP=\frac{\sum AP}{N\left(classes\right)}$$

where AP is the average correct rate, which represents the result of good or bad detection for each class.

mAP0.5 means that the value of IoU is taken as 50%. mAP0.5:0.95 means that the value of IoU is taken from 50% to 95% in steps of 5%, and then the mean value of mAP under these IoUs is calculated.

There are two ways to define small targets: one is a relative size, such as a target size with a length and width of 0.1 of the original image size, which can be considered a small target; the other is the absolute size, which means a target size less than 32 × 32 pixels can be considered a small target. In this article, we define small targets as those using absolute dimensions, smaller than 32 × 32 pixels.

### 4.4. Experimental Results

This section describes experiments conducted using the YOLOv8 model as the baseline, with both ablation and comparison experiments performed. Specifically, the MLCA (mixed local channel attention) was added to the C2f module of YOLOv8, and the neck part of YOLOv8 was redesigned by incorporating SSFF (scale sequence feature fusion) and TFE (triple feature encoding) into the neck. Additionally, the P2 layer was added to enhance the detection of small objects.

#### 4.4.1. Model Validation

**Figure 9:**
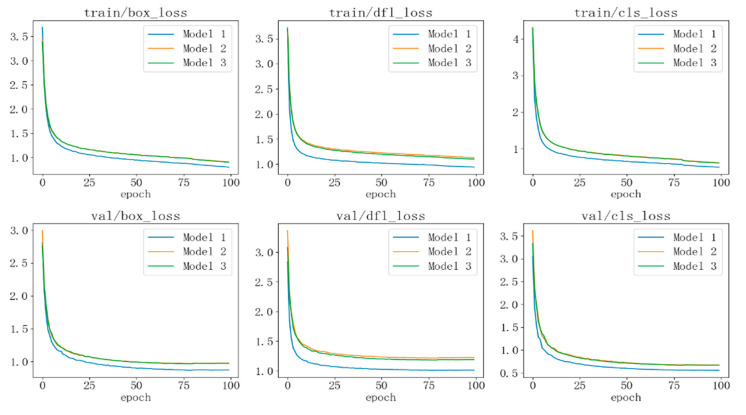
Model training and validation loss curves.

Before starting the model training, the initial learning rate was set to 0.01, and the weight decay coefficient was set to 0.0005. The batch size was set to 16, and the input image size was uniformly adjusted to 640 × 640. The number of threads during data loading was set to 8, and the system automatically selected the most suitable optimizer based on the characteristics of the model and training task. Mosaic and Mixup data augmentation strategies were applied, with Mosaic disabled during the last 20 training epochs. Training was conducted using automatic mixed precision (AMP) [26], with the total number of training epochs set to 100. Model1 refers to the original YOLOv8 model, Model2 incorporates the ST-P2Neck module, and Model3 integrates both the ST-P2Neck and C2f-MLCA modules. As shown in Figure 9, both the training loss and validation loss steadily decreased and eventually converged to their minimum values, with no divergence or overfitting observed, effectively demonstrating the rationality of the improvements made to the YOLOv8 model.

#### 4.4.2. Ablation Experiment

The performance of the model was tested on the SODA-10M dataset, with the test results shown in Table 1 where the “√” symbol indicates the corresponding method applied in each model. After adding the ST-P2Neck module, compared with the original YOLOv8 model, the precision (P) improved by 2.64%, recall (R) improved by 7.82%, and mAP_0.5 increased by 7.53%. This demonstrates that the model’s performance was effectively enhanced with the inclusion of the ST-P2Neck. Furthermore, when the C2f-MLCA module was added on top of the ST-P2Neck, the precision increased by 3.43%, recall by 8.15%, and mAP_0.5 by 8.42%, further confirming the effectiveness of incorporating both the ST-P2Neck and C2f-MLCA modules.

With the addition of these modules, the parameter count decreased and the computational load slightly increased, so we conducted practical testing on them. The preprocess of the original YOLOv8 model for practical detection in unmanned driving scenarios is 1.2 milliseconds, the inference is 8.0 milliseconds, and the post-process is 6.8 milliseconds. The MST-YOLOv8 model has a preprocessing time of 1.3 milliseconds, an inference time of 10.1 milliseconds, and a post-processing time of 8.4 milliseconds during actual testing. Although the detection speed has slightly decreased, real-time performance can still be maintained while improving detection accuracy.

**Figure 10:**
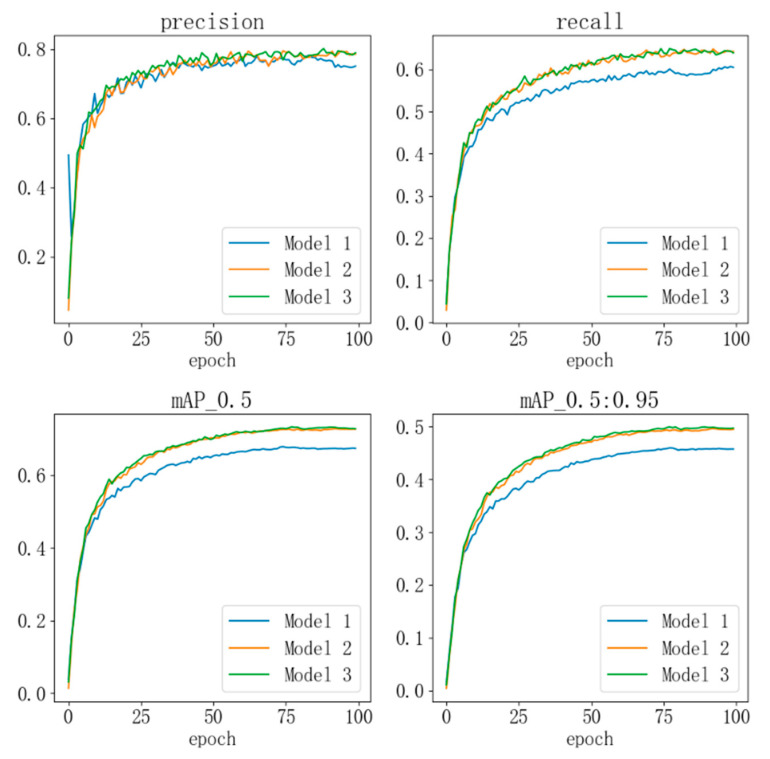
Model performance during training.

**Figure 11:**
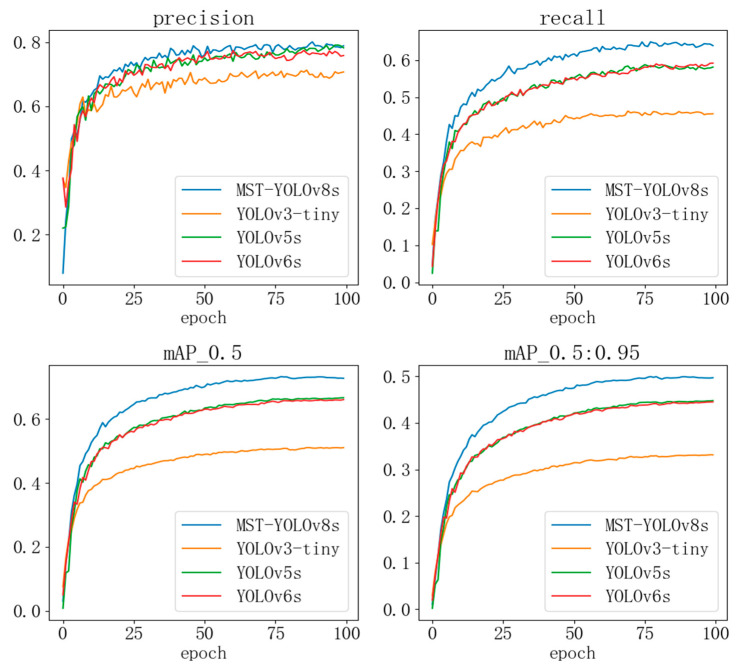
Comparison of experimental model performance.

Figure 10 illustrates the improvement in model performance during the training process. The curves are smooth, indicating the model’s stability without significant fluctuations. The blue curve represents the original YOLOv8 model, the yellow curve corresponds to the model with the ST-P2Neck module, and the green curve represents the model incorporating both the ST-P2Neck and C2f-MLCA modules. As seen in Figure 3, Figure 4, Figure 5, Figure 6, Figure 7, Figure 8, Figure 9, Figure 10 and Figure 11, the model with the ST-P2Neck and C2f-MLCA modules demonstrates a clear improvement in precision (P), recall (R), and mAP values compared with the original YOLOv8 model.

As shown in Table 2, after incorporating the ST-P2Neck and C2f-MLCA modules, both the localization error rate and the miss detection rate of the model have decreased. Additionally, the AP and AR values for small object detection have improved (with IoU = 0.50:0.95 for AP and AR), demonstrating the effectiveness of the model enhancements.

#### 4.4.3. Comparison Experiments

To validate the rationality of the model improvements, comparative experiments were conducted with several other models from the YOLO series. The experimental results are shown in Figure 11. The blue curve represents the MST-YOLOv8 model, the yellow curve is YOLOv3-tiny, the green curve is YOLOv5s, and the red curve is YOLOv6s. As seen from the curves, the MST-YOLOv8 model outperforms the other models in terms of precision, recall, and mAP, demonstrating the effectiveness of the model improvements.

We also conducted comparative experiments with other small object detection models, and the experimental results are shown in Table 3.

**Figure 12:**
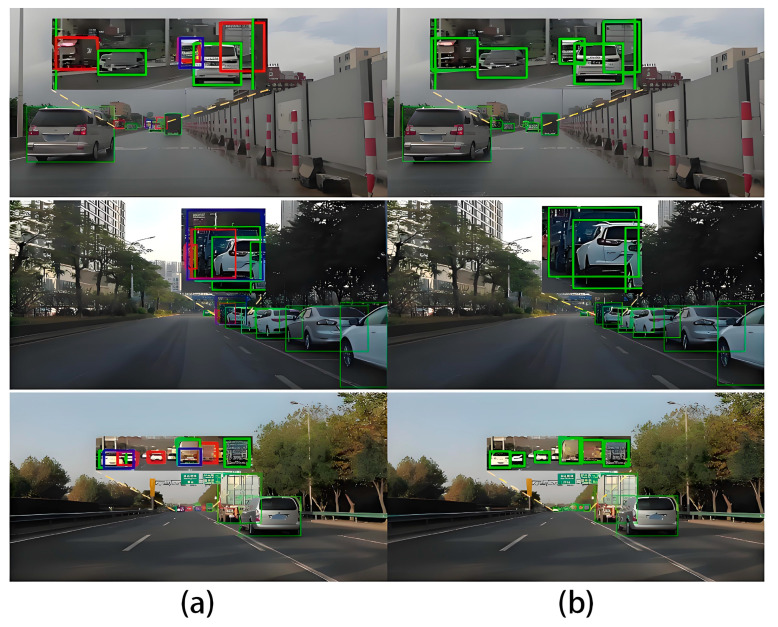
Model detection effect comparison chart. (**a**) These images represent the output of the YOLOV8 model; (**b**) these images represent the output of MST-YOLO. Red represents missed detection, blue represents false detection. Green represents the correct detection of the target to be detected.

To verify the detection performance of the model, the trained model was tested on the SODA-10M test set, and the results were visualized. As shown in Figure 12, the left image represents the detection results from the MST-YOLOv8 model, while the right image shows the results from the original YOLOv8 model. The detected bounding boxes were compared with the original labels. Green boxes indicate True Positives, meaning the model correctly predicted positive samples. Blue boxes represent False Positives where the model incorrectly predicted negative samples as positive. Red boxes denote False Negatives where the model failed to detect positive samples. If the same object is outlined by both a red and a blue box, it indicates that the label was detected but classified incorrectly.

As shown in Figure 12, the MST-YOLOv8 model demonstrates superior performance in detecting small objects, while the original YOLOv8 model exhibits instances of both missed detections and false positives. This comparison highlights the effectiveness of the improvements made in the experiment.

**Figure 13:**
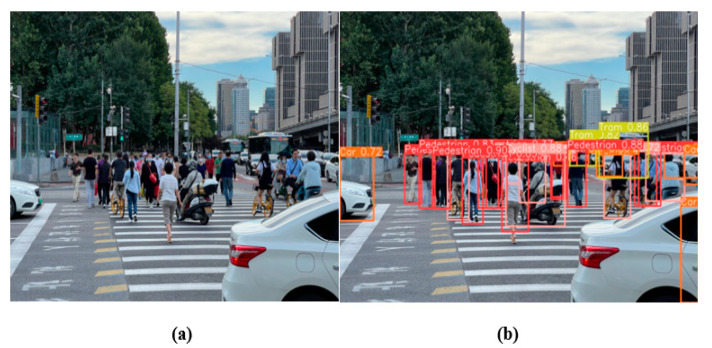
The detection effect of the model under dense traffic flow conditions. (**a**) This image represents the original image; (**b**) this image represents the output of MST-YOLO.

As shown in Figure 13, the detection performance of the MST-YOLO model under dense traffic flow is demonstrated.

**Figure 14:**
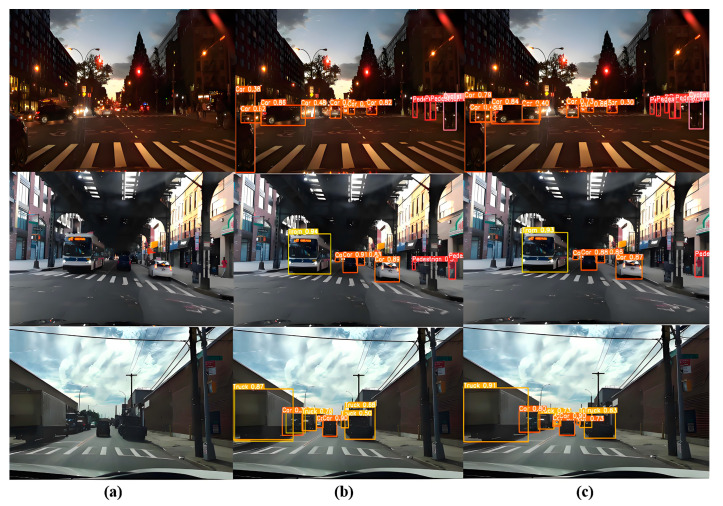
The detection performance of the MST-YOLOv8 model on the BDD100K dataset: (**a**) the original image, (**b**) the result output by YOLOV8; (**c**) the result output by MST-YOLO.

To validate the model’s generalization capabilities, we selected 1000 images from the BDD100K dataset for real-world detection testing. The results, shown in Figure 14, include three distinct autonomous driving scenarios: sunny conditions, occluded lighting, and evening scenes. The detection targets consist of five categories: pedestrian, cyclist, car, truck, and tram. It is evident that the MST-YOLOv8 model is less affected by factors such as lighting and occlusion, adapting well to various human–vehicle scenarios in autonomous driving and showing strong detection performance across different object scales.

## 5. Conclusions

In this paper, an MST-YOLOv8 model is designed based on the original YOLOv8 architecture. The improved C2f structure, named C2f-MLCA, incorporates the mixed local channel attention (MLCA) into the C2f module of YOLOv8. The improved neck is named ST-P2Neck, which is a redesigned neck part of YOLOv8, integrating scale sequence feature fusion (SSFF) and triple feature encoding (TFE) into the neck and adding a P2 layer to enhance small object detection. Compared with the original YOLOv8, the MST-YOLOv8 model shows improvements in precision (P) by 3.43%, recall (R) by 8.15%, and mAP_0.5 by 8.42%. Moreover, it reduces the miss detection rate for small objects, improving the accuracy of object detection in autonomous driving and, thereby, enhancing safety in autonomous vehicle applications.

## Figures and Tables

**Table 1:** Model performance comparison.

	ST-P2Neck	C2f-MLCA	P	R	mAP_0.5	Param	FLOPs
Model 1			0.758	0.601	0.677	11.13M	28.4G
Model 2	√		0.778	0.648	0.728	9.05M	35.8G
Model 3	√	√	0.784	0.65	0.734	9.05M	35.8G

**Table 2:** Comparison of model missed detection rate and false detection rate.

	ST-P2Neck	C2f-MLCA	E_loc_↓	E_miss_↓	AP_small_↑	AR_small_↑
Model 1			5.62	2.87	0.124	0.251
Model 2	√		3.50	2.31	0.209	0.423
Model 3	√	√	3.13	2.34	0.212	0.424

**Table 3:** Comparison with other small object detection models.

Model	P	R	mAP_0.5	Param	FLOPs
Efficientdet-d1	0.742	0.582	0.663	6.45M	21.4G
RetinaNet	0.757	0.607	0.676	8.13M	27.6G
Ours	0.784	0.65	0.734	9.05M	35.8G

## Data Availability

Data are contained within the article.
